# The generation and evaluation of recombinant human IgA specific for *Plasmodium falciparum *merozoite surface protein 1-19 (*Pf*MSP1_19_)

**DOI:** 10.1186/1472-6750-11-77

**Published:** 2011-07-22

**Authors:** Jianguo Shi, Richard S McIntosh, Jaime Adame-Gallegos, Prabhjyot K Dehal, Marjolein van Egmond, Jan van de Winkel, Simon J Draper, Emily K Forbes, Patrick H Corran, Anthony A Holder, Jenny M Woof, Richard J Pleass

**Affiliations:** 1 Institute of Genetics, Queen's Medical Centre, University of Nottingham, NG7 2UH, UK; 2 Division of Medical Sciences, University of Dundee Medical School, Ninewells Hospital, Dundee, DD1 9SY, UK; 3 Department of Molecular Cell Biology and Immunology, VU Medical Centre, Amsterdam, 3508 TC, The Netherlands; 4 Immunotherapy laboratory, Department of Immunology, University Medical Centre Utrecht, Utrecht, 3508 TC, The Netherlands; 5 The Jenner Institute, University of Oxford, Oxford, OX3 7DQ, UK; 6 Department of Infectious and Tropical Diseases, London School of Hygiene and Tropical Medicine, London, WC1E 7HT, UK; 7 Division of Parasitology, National Institute for Medical Research, London, NW7 1AA, UK

## Abstract

**Background:**

Human immunoglobulin G (IgG) plays an important role in mediating protective immune responses to malaria. Although human serum immunoglobulin A (IgA) is the second most abundant class of antibody in the circulation, its contribution, if any, to protective responses against malaria is not clear.

**Results:**

To explore the mechanism(s) by which IgA may mediate a protective effect, we generated fully human IgA specific for the C-terminal 19-kDa region of *Plasmodium falciparum *merozoite surface protein 1 (*Pf*MSP1_19_), a major target of protective immune responses. This novel human IgA bound antigen with an affinity comparable to that seen for an epitope-matched protective human IgG1. Furthermore, the human IgA induced significantly higher NADPH-mediated oxidative bursts and degranulation from human neutrophils than the epitope-matched human IgG1 from which it was derived. Despite showing efficacy in *in vitro *functional assays, the human IgA failed to protect against parasite challenge *in vivo *in mice transgenic for the human Fcα receptor (FcαRI/CD89). A minority of the animals treated with IgA, irrespective of FcαRI expression, showed elevated serum TNF-α levels and concomitant mouse anti-human antibody (MAHA) responses.

**Conclusions:**

The lack of protection afforded by MSP1_19_-specific IgA against parasite challenge in mice transgenic for human FcαRI suggests that this antibody class does not play a major role in control of infection. However, we cannot exclude the possibility that protective capacity may have been compromised in this model due to rapid clearance and inappropriate bio-distribution of IgA, and differences in FcαRI expression profile between humans and transgenic mice.

## Background

There is increasing interest in exploring the therapeutic potential of alternative antibody (Ab) classes to IgG, which to date has been the most popular choice, with over 160 examples currently in clinical trials for the treatment of diverse cancers, infectious diseases and auto-immune conditions [1,2]. We recently developed a novel humanized mouse model to show that human IgG1 specific for *Plasmodium falciparum *merozoite surface protein 1-19 (*Pf*MSP1_19_) could protect animals from malaria in passive transfer experiments [3]. However there are numerous drawbacks to using IgG-based therapies in malaria, including competition for FcR binding, from high levels of parasite-induced non-specific IgG [4], that warrant the exploration of other serum Ab classes for use against infections of blood.

FcαRI (CD89) targeting with IgA could offer potential for controlling malaria with therapeutic antibodies [5]. Unlike IgM, IgG and IgE, which are implicated in immune evasion [6], placental malaria [7] and severe malaria respectively [8], IgA has not been implicated in malaria pathology, arguing for its consideration in Ab therapy. Although a direct role for murine IgA in killing of rodent malaria parasites has not been investigated *in vivo *because mice lack an equivalent of human FcαRI, *Plasmodium*-specific IgA has been detected at high levels in serum [9,10], and breast milk [10,11], in humans from endemic areas.

Ligation of the myeloid FcαRI induces cytokine release and can stimulate a respiratory burst [12,13], and FcαRI is better than FcγRs at triggering lysis of Ab-targeted tumors as well as phagocytosis of pathogens coated with Abs, both in humans and mice [13,14]. Human FcαRI is expressed on the majority of white blood cells, including neutrophils, monocytes, macrophages, eosinophils, platelets and NK cells, suggesting it to be an ideal target for systemic IgA-mediated therapy [4,5,13,15,16]. The finding that FcαRI is a discrete modulator of the immune system mediating both anti- and pro-inflammatory functions indicates that further exploration of the role of human IgA in malaria is necessary [17]. We recently described a mandatory role for human FcαRI in mediating protection from tuberculosis using recombinant human IgA [18].

To address the role of human IgA in malaria, we generated a recombinant IgA recognizing the *Pf*MSP1_19 _epitope, matched to a human IgG1 shown previously to transfer passive protection in the FcγRI (CD64) transgenic mouse model [3]. This recombinant IgA was then tested in passive transfer experiments for efficacy in controlling malaria *in vivo *in human FcαRI (CD89) transgenic mice.

## Results

### 1. Characterization of *Pf*MSP1_19_-specific human IgA

**Figure 1:**
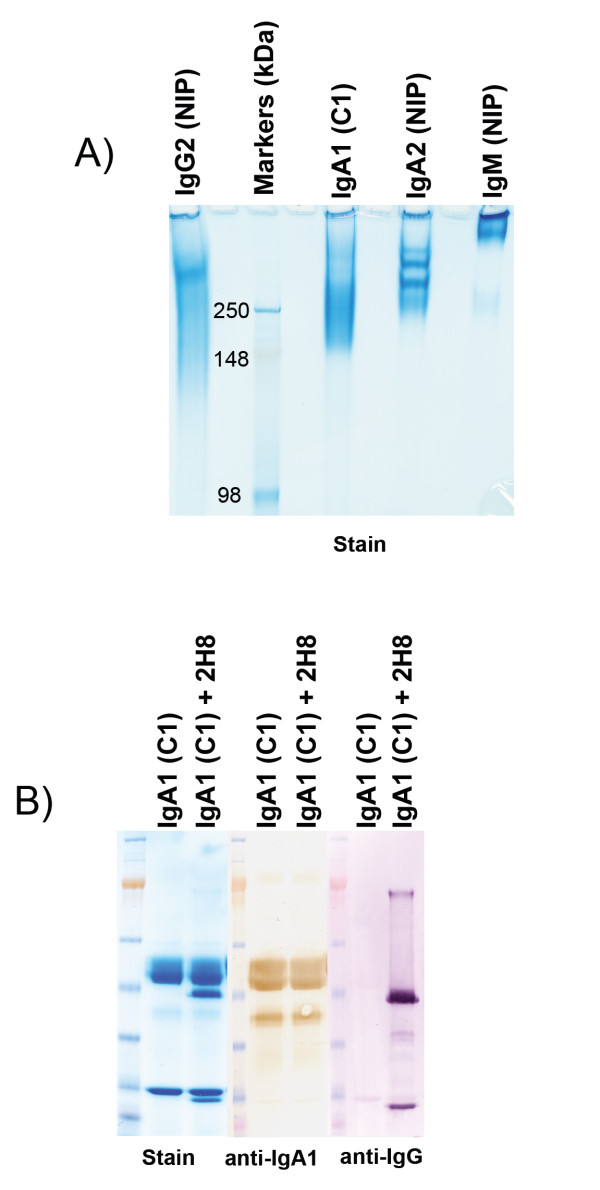
**Characterization of purified recombinant *Pf*MSP1_19_-specific human IgA**. 5 μg purified recombinant Abs were run under (**A**) non-denaturing (native) conditions on 6% acrylamide Tris-glycine gels (Novex) or under (**B**) denaturing reducing conditions on NuPAGE 4-12% Bis-Tris gradient SDS gels (Novex). In the former, anti-NIP IgA2 (AbD Serotec, Kidlington, UK) and anti-NIP IgM (AbD Serotec) were included as polymeric controls. In the latter, gels were either stained with Coomassie or transferred to nitrocellulose and probed with anti-human IgA or anti-mouse IgG-Fc reagents, and lanes containing both IgA and the anti-FcαRI mAb 2H8 were included. The IgA is recognized by anti-human IgA-specific reagents but not by anti-mouse IgG-Fc reactive Abs that only recognize mAb 2H8.

*Pf*MSP1_19_-specific human IgA isolated from HEK-293T transfectant culture supernatants by affinity-chromatography appeared pure (Figure 1). It ran ahead of recombinant anti-NIP polymeric IgA2 and IgM under native conditions (Figure 1A), suggesting that it is mainly monomeric, a conclusion supported by size-exclusion chromatographic analysis (data not shown). The anti-NIP antibodies are in polymeric form having been produced in J-chain expressing transfectants. On reducing SDS-PAGE gels, the recombinant IgA resolved into heavy and light chain bands of the anticipated molecular mass (Figure 1B). The heavy chain was recognized by an isotype-specific Ab. The recombinant human IgA recognized a *P. falciparum *MSP1_19_-GST fusion protein in ELISAs and by indirect IFA produced a characteristic pattern of MSP1 reactivity in schizonts, merozoites, and ring-stage parasites from *P. falciparum *and *Plasmodium berghei *parasites transgenic for *Pf*MSP1_19 _(data not shown). Importantly, surface plasmon resonance (SPR) analysis revealed no reduction in affinity for *Pf*MSP1_19 _when compared with the parental IgG1 antibody (Table 1). The binding constants remain essentially the same, and although the affinity for *Pf*MSP1_19 _is less than that of a well characterized mouse monoclonal antibody (mAb) 12.10 specific for *Pf*MSP1_19 _(Table 1), it is still appreciable.

**Table 1:** Analysis of the binding properties of *Pf*MSP1_19_-specific human IgA (C1), human IgG1 (C1) and mouse mAb 12.10 to immobilized GST-*Pf*MSP1_19 _by SPR.

	**K**_**ass**_	**K**_**diss**_	**K**_**d**_
	(M^-1 ^sec^-1^)	(sec^-1^)	(M)
mAb 12.10	5.37 × 10^5^	5.5 × 10^-6^	1 × 10^-11^
IgG1 (C1)	4.4 × 10^3^	1.0 × 10^-4^	2.3 × 10^-8^
IgA (C1)	6.5 × 10^3^	1.7 × 10^-4^	2.6 × 10^-8^

(K_d _= K_diss_/K_ass_)

Methodology described in refs [3,13].

### 2. IgA1 triggers *Pf*MSP1_19_-specific neutrophil nicotinamide adenine dinucleotide phosphate (NADPH) oxidase activation through FcαRI cross-linking

**Figure 2:**
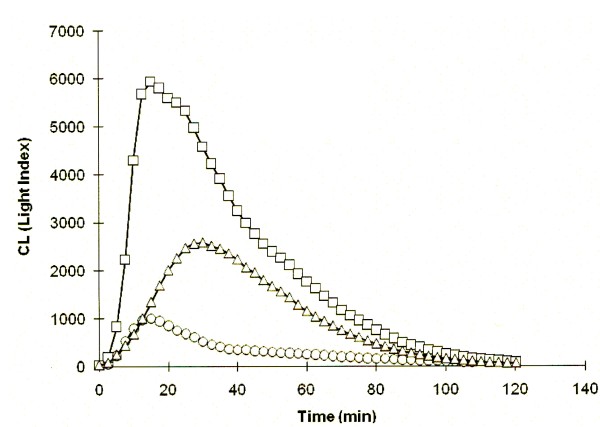
**Recombinant human *Pf*MSP1_19_-specific IgA is functional**. Stimulation of neutrophil NADPH oxidative bursts using 50 μg of IgA attached to GST-*Pf*MSP1_19_-coated microtiter plates (open squares), or 50 μg of IgA attached directly to the bottom of the plate (open triangles) or no Ab (open curcles). Data are presented as mean chemiluminescence (CL; arbitary units) from triplicate wells using neutrophils from a single human donor as previously described [3,12,13].

We next assessed the ability of this novel IgA to interact with human FcαRI and induce NADPH oxidase activation (respiratory burst) and degranulation in human blood neutrophils (Figure 2). For neutrophils, luminol chemiluminescence provides a read out of NADPH oxidase activation and myeloperoxidase release [12,13]. When attached to *Pf*MSP1_19_-GST-coated plates, the human IgA induced significantly greater respiratory bursts than if the IgA was coated directly to the bottom of the plate in the absence of antigen (Figure 2), suggesting that binding to antigen allows the IgA to be presented to neutrophil FcαRI in an optimal configuration for receptor cross-linking and triggering of functional responses. We have observed that this IgA induces greater respiratory bursts from human neutrophils than the epitope-matched human IgG1 from which this Ab was derived [3]. This ability of human IgA to outperform human IgG1 at inducing free oxygen radicals and activation of human neutrophils has also been observed for human IgA recognizing the homologous antigen from *Plasmodium yoelii *[13].

### 3. Passive transfer of human IgA into wild type or human FcαRI (CD89) transgenic mice has no effect on the course of a malaria infection

**Figure 3:**
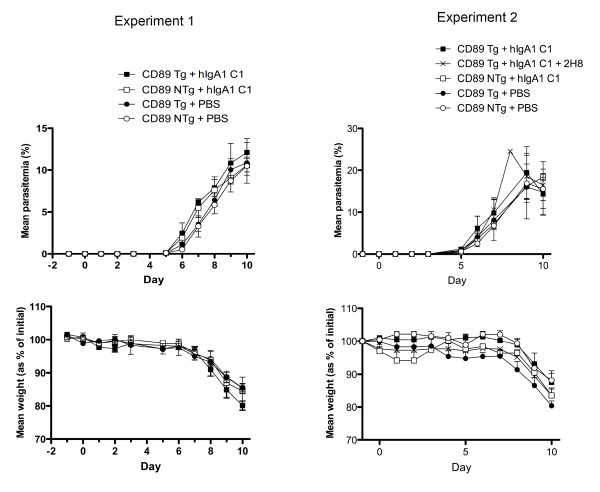
**Course of a *Pf*MSP1_19 _transgenic *P. berghei *infection in mice**. Groups of 3 (experiment 1) or 4 (experiment 2) FcαRI transgenic (Tg) or nontransgenic (NTg) littermates were injected *i.p*. with a total dose of 1.5 mg human anti-*Pf*MSP1_19 _IgA (C1) or phosphate buffered saline (PBS). No significant differences were observed for either the course of parasitemia or weight loss in both experiments. Each point represents the geometric mean parasitemia or weight of mice in each group at the time after *i.p*. challenge with 5,000 parasitized erythrocytes. In experiment 2, the FcαRI-blocking mAb 2H8 was co-administered at the same time as the IgA into CD89 Tg animals.

Because FcαRI is absent in mice due to a translocation event in the leukocyte receptor complex, we tested the ability of the human IgA to protect from malaria using human FcαRI transgenic mice [19,20]. These mice have been used to show protection from mucosally administered pathogens but have not been directly investigated in relation to blood parasites or in the context of human IgA administered systemically [21,22]. Blood granulocytes and a proportion of monocytes from these transgenic mice constitutively express functional human FcαRI and bind human IgA (Additional file 1, Figure S1). *In vivo *experiments with *P. berghei *transgenic for *Pf*MSP1_19 _highlighted that three intraperitoneal (*i.p*.) inoculations of IgA (total dose of 1.5 mg Ab per mouse) was unable to suppress a lethal blood stage challenge infection in these animals in contrast to previous observations with the epitope-matched human IgG1 passively administered into human FcγRI (CD64) transgenic animals [3] (Figure 3).

### 4. Passive transfer of *Pf*MSP1_19_-specific human IgA1 into mice induces TNF-α and mouse anti-human antibody (MAHA) responses in some animals

**Figure 4:**
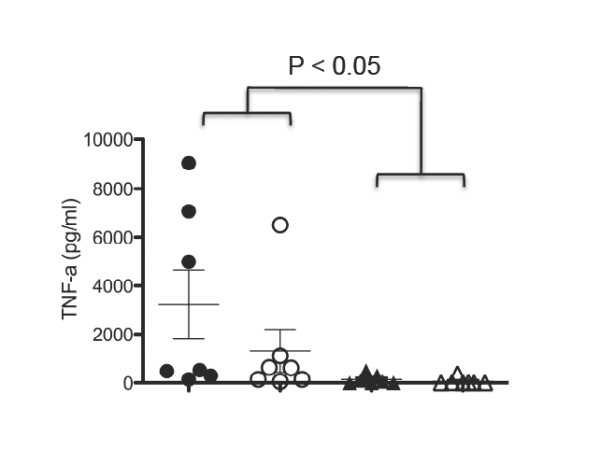
**Plasma TNF-α responses in animals given *i.p*. *Pf*MSP1_19_-specific IgA**. Each bar represents the mean concentration of TNF-α ± SE from each group of mice in Fig. 3. Levels of TNF-α are shown for CD89 Tg (Tg) animals passively transferred with human IgA (closed circles) or PBS (open circles) against non-transgenic (NTg) littermates also given IgA (closed triangles) or PBS (open triangles). Each plasma sample was assayed in triplicate determinations for each animal. Differences between the groups were analyzed using Dunn's multiple comparison test and statistically significant differences are indicated.

We observed that animals passively immunised with *Pf*MSP1_19_-specific human IgA by the *i.p*. route showed greater adverse clinical signs according to the Laboratory Animal Science Association (LASA) indices, including severe piloerection & lethargy, after challenge with malaria parasites than control groups of animals. To investigate the underlying basis for these observed detrimental effects of the passively transferred human IgA we analyzed serum cytokines responses in all the animals using the cytometric bead array mouse Th1/Th2 10plex kit (Bender Medsystems) on day 10 post-challenge when all mice were killed. We observed significantly elevated levels of TNF-α in animals treated with human IgA compared with control animals given PBS, irrespective of genetic background (Figure 4), although extreme levels of TNF-α were only seen in four of the fourteen mice tested. The difference in TNF-α levels between the transgenic and non-transgenic groups administered IgA was not statistically significant (Figure 4). No significant differences over baseline levels of IL-4, IL-10, IFN-γ, IL-6, IL-17, GM-CSF were observed between the groups, although IL-2 and IL-1α were raised in animals given IgA when compared with PBS (data not shown). Co-administration of mAb 2H8, a mouse mAb that binds to human FcαRI blocking the human IgA binding site [23], at the time of IgA delivery, failed to dramatically alter the course of parasitemia (Figure 3) or the development of TNF-α responses (not shown). The induction of elevated TNF-α was therefore not dependent on the presence of the human FcαRI transgene, but more likely due to some uncharacterized effect of the *Pf*MSP1_19_-specific IgA, a result supported by the raised TNF-α levels observed in non-transgenic animals also given the antibody. These high levels of TNF-α were present despite the absence of detectable *Pf*MSP1_19_-specific IgA in the final bleeds (in contrast to similar experiments with IgG1), confirming that the half-life of the passively transferred human IgA in mice is less than that for human IgG1. All animals given *Pf*MSP1_19_-specific IgA (irrespective of genetic background), also made significant mouse anti-human IgA responses on day 10 as assessed by ELISA (Additional file 1, Figure S2), suggesting that the high levels of inflammatory cytokines observed may be a consequence of mounting an anti-human IgA immune response and are not the result of malaria infection *per se*, as these cytokines were not observed in challenged mice given PBS.

## Discussion

We describe the development of a fully human IgA with specificity for a very well characterized epitope on MSP1_19 _from *P. falciparum*, useful for dissecting human Fc receptor mechanisms involved in immunity to human malaria [3].

This novel IgA was generated from a human IgG1 mAb (C1), previously shown to protect human FcγRI (CD64) transgenic mice (but not wild type animals) from a lethal challenge with rodent malaria (*P. berghei*) transgenic for the *P. falciparum *MSP1_19 _(*Pf*MSP1_19_) antigen [3]. The engineered IgA recognized parasites in infected erythrocytes and bound *Pf*MSP1_19 _with comparable kinetics to that observed with the parental IgG1 from which it was derived. Despite triggering potent and functional *in vitro *responses the IgA failed to protect human FcαRI transgenic mice from a malaria infection. Although the injected IgA is mostly monomeric, and therefore binds transiently to FcαR, immune-complexes formed on engaging *Pf*MSP1_19 _on merozoites would be expected to bind avidly to FcαRI [1,2]. We also tried without success to passively protect the CD89 transgenic animals with sera from rabbits immunized with *Pf*MSP1_19 _or plasma from vaccinated humans or humans from endemic regions with significant levels of anti-*Pf*MSP1_19 _human IgA antibodies by ELISA (data not shown). This is noteworthy, as clinical protection from *P. falciparum *malaria, has recently been shown to correlate with neutrophil respiratory bursts induced by merozoites opsonized with human serum antibodies [24]. This important study demonstrated that immune African sera depleted of IgG by protein-G Sepharose chromatography (but presumably still containing IgA), gave negligible activity in their antibody-dependent respiratory burst assays (ADRB). Whilst highlighting an absolute requirement for IgG, this finding is however difficult to reconcile with the observed ability here of *Pf*MSP1_19_-specific human IgA to induce very effective luminol NADPH-oxidase mediated respiratory bursts and degranulation from human blood neutrophils (Figure 2) [12,13].

One explanation for the failure of the IgA to protect against malarial challenge may be that it is difficult for IgA administered *i.p*. to leave the peritoneum and reach the circulation and other parts of the body, where any protective effects would presumably be mediated. Transfer of IgG from peritoneum to other body compartments may be assisted, at least in part, through interaction with FcRn, an IgG receptor present on a variety of cell types, capable of bidirectional transport across epithelial surfaces and involved in control of IgG turnover [25]. IgA, in contrast, is unable to bind FcRn, and may be more reliant on diffusion to reach the circulation and distribute within other body compartments. This may also explain why recombinant IgA when administered intra-nasally was particularly effective at controlling tuberculosis [18].

Another explanation for the lack of *in vivo *efficacy may be that the administered IgA was cleared very rapidly from the mouse, before it had sufficient opportunity to mediate any protective effects. Unlike humans, where the half-life of the chiefly monomeric serum IgA is approximately 4-5 days [26], in mice monomeric IgA has a half-life of just 24 hours [27]. IgG is cleared much more slowly due to interaction with FcRn, and therefore when administered *i.p*. presumably has a much greater opportunity for functional impact.

A third possibility is that the FcαRI in the transgenic mice is not expressed on the cell types critical for protection at sufficiently high levels, or on a sufficient proportion of these cells, or on cells located within the required body compartment. For example, in contrast to the situation in humans, only a subpopulation of the monocytes of the transgenic mice express human FcαRI, and peritoneal macrophages and platelets exhibit no expression [19].

These three possibilities, taken together, suggest that the FcαRI transgenic mouse, although undoubtedly the best model currently available, may not afford the means to fully assess the capability of human IgA to offer protection in the human setting.

This is the first recombinant human IgA to target *Pf*MSP1_19_. However, in exploring the role of IgA in malaria, others have tested recombinant human IgA1 and IgA2 localized on the surface of polystyrene beads in a two-step antibody-dependent cellular inhibition (ADCI) assay [28]. In contrast to human IgG1 and IgG3, neither IgA1 or IgA2 were found to stimulate *in vitro *ADCI of malaria parasites by human monocytes [28]. Human monocytes express FcαRI and induce parasite inhibitory TNF-α [29], and may therefore have been expected to engage in ADCI. This finding is in keeping with the lack of an *in vivo *effect seen here, the data suggesting neutrophils rather than monocytes may fulfill this role, and with either human IgA targeting the *P. berghei Pf*MSP1_19 _transgenic, or earlier against *P. yoelii *MSP1_19 _[13]. However, it will be necessary to generate epitope-matched murine IgAs (and IgGs) to determine if mouse monocytes/neutrophils are capable of ADCI/ADRB and whether mouse IgA can protect against rodent malarias *in vivo*. Although no counterpart for human FcαRI is known in mice, mouse macrophages do bind mouse IgA, and Mac-2/galectin-3 (gal-3) has been suggested to be the receptor involved [30]. Interestingly, gal-3 is known to bind IgE, and there may be some mileage in developing recombinant IgE to investigate malaria infection, as mouse IgE can also bind FcγRIV (which also binds IgG2a and IgG2b) on mouse monocytes [31]. Intriguingly, murine IgE has been shown to confer protection from *P. berghei *in C57BL/6 mice, and elevated anti-malarial IgE in asymptomatic individuals has been shown to associate with a reduced risk of subsequent clinical malaria in humans [32].

We observed that a small proportion of mice given the recombinant human IgA preparation in the context of a malaria infection developed significant mouse anti-human antibody responses (MAHA) with elevated TNF-α within 10 days of the last IgA dose, which were not observed in control animals challenged with malaria (Figure 4). Our observations suggest that the responses are not dependent on the presence of the FcαRI transgene and therefore may represent a reaction to the administered IgA itself. Further experiments will be necessary to clarify whether the response noted is peculiar to the particular IgA preparation used which may contain undetected levels of aggregates, or the specificity of the IgA for the co-administered malarial parasite that might result in particular cross-linking events, or is a general effect associated with human IgA administration to mice in the absence of malaria challenge infection.

## Conclusions

In summary, a novel *Pf*MSP1_19_-specific IgA did not show protective capability against parasite challenge in mice transgenic for human FcαRI. While this finding may indicate that IgA does not play a major role in protection against malaria, we cannot rule out the possibility that the findings reflect certain shortcomings of the mouse model used. Thus important differences in IgA half-life, IgA bio-distribution, and FcαRI expression profile between the transgenic mice and humans may have compromised the antibody's ability to mediate protective effects, and further experimentation will be required to fully dissect the role of IgA in human malaria.

## Methods

### Ethics Statement

Informed written consent was obtained from all participants and approval for the use of human samples was obtained from the Ethical Committees at Nottingham and Oxford. All animal experiments were approved by the Home Office and performed in accordance with UK guidelines and regulations (PPL 40/2753).

### Construction of human IgA

The variable heavy (VH) gene from pVH-C1-γ1 was subcloned as a BssHII/BstEII fragment upstream of the human IgA1 α-chain constant region sequence previously inserted into the mammalian expression vector pcDNA3.1/Hygro (Invitrogen, UK), to create pVH-C1-α1. Mammalian HEK293T cells (European Collection of Cell Cultures) were co-transfected with linearized pVH-C1-α1 and pVK-C1-Express (expression vector containing the corresponding C1 variable light (VL) chain upstream of the human Cκ gene). Positive clones secreting *Pf*MSP1_19_-specific IgA were detected by enzyme-linked immunosorbent assay (ELISA) with recombinant *Pf*MSP1_19 _coated plates and by immunoblotting with goat anti-human IgA Abs conjugated to horseradish peroxidase (HRP) or alkaline phosphatase (AP) as previously described [3,13]. Recombinant *Pf*MSP1_19 _used in all ELISAs was generated as previously described [33]. From large-scale cultures, human IgA was purified by affinity chromatography on anti-human IgA agarose columns by FPLC (Sigma). The integrity and purity of the antibodies was verified by gel electrophoresis on both non-denaturing and denaturing gels according to the manufacturer's instructions (Novex) (see Figure 1).

### Luminol chemiluminescence assay of respiratory burst and myeloperoxidase release

Neutrophils were isolated from heparinized blood taken from healthy volunteers by sedimentation of erythrocytes in 6% (w/v) dextran T70 (GE Healthcare, U.K.) in 0.9% (w/v) saline at 37°C for 30 min, followed by leukocyte separation on a discontinuous density gradient of Lymphoprep (ρ = 1.077 g/cm^3^; Nycomed, Birmingham, UK) over Ficoll-Hypaque (ρ = 1.119 g/cm^3^), centrifuged at 700 × *g *for 20 min at room temperature. Approval for the collection and use of human cells was obtained from the local Queen's Medical Centre ethical committee. Wells of chemiluminescence microtiter plates (Dynatech Laboratories, Billinghurst, Sussex, UK) were coated with 150 μl of *Pf*MSP1_19 _at 5 μg ml^-1 ^or 50 μg of IgA in coating buffer (0.1 M carbonate buffer, pH 9.6) and incubated overnight at 4°C. After washing three times with PBS, 150 μl of anti-*Pf*MSP1_19 _IgA at 50 μg ml^-1 ^was added to antigen coated wells. In each case, triplicate wells were prepared and left for 2 h at room temperature. After washing as before, 100 μl of luminol [67 μg ml^-1 ^in Hank's buffered saline solution (HBSS) containing 20 mM HEPES and 0.1 g/100 ml globulin-free BSA (HBSS/BSA)] were added to each well. After the addition of 50 μl of purified neutrophils (10^6^/ml in HBSS/BSA) to each well, plates were transferred to a Microlumat LB96P luminometer, and the chemiluminescence measured at 2 min intervals for 120 min at 37°C. Data were analyzed using Excel software.

### Cytokines

Concentrations of GM-CSF, IFN-γ, IL-1α, IL-2, IL-4, IL-5, IL-6, IL-10, IL-17 and TNF-α in individual mouse sera were determined by flow cytometry using the FlowCytomix mouse Th1/Th2 10plex (BMS820FF) bead kit against known standard curves and according to manufacturer's instructions (Bender MedSystems). Beads were analyzed using a Beckman Coulter EPICS ALTRA flow cytometer (High Wycombe, Bucks, UK) and data analyzed with Bender MedSystems software.

### Passive immunization and parasite challenge

Because of the lack of an animal model for *P. falciparum *and because mice do not express a homologue of human FcαRI, human FcαRI transgenic mice have been developed that express FcαRI on blood neutrophils and a proportion of their monocytes [Additional file 1, Figure 1 [22]]. Transgenic (Tg) Balb/c × Balb/c F1 mice 9-12 wks old and bred under specific pathogen-free conditions were used. Non-transgenic (NTg) littermates served as controls. Mice were screened for FcαRI expression by PCR of whole blood using forward (5'-TGGGGCTTCGCACAGGGTCTTTA-3') and reverse (5'-CCAGCACACCGCAGTCGCCATAC-3') primers for human CD89, and by analysis of lysed whole blood on a FAC-Scan with PE-conjugated anti-human FcαRI (Additional file 1). *Pf*MSP1_19_-specific IgA (at 0.5 mg/injection) with or without 50 μg/injection blocking mAb 2H8 (mouse IgG1 anti-human FcαRI) [23] was administered intraperitoneally (*i.p*.) on days -1, 0 and day +1 with respect to parasite challenge. Parasitized erythrocytes (5000/mouse) derived from passaged mice infected with *P. berghei *parasites transgenic for *P. falciparum *MSP1_19 _were injected *i.p*. at least 3 h after antibody treatment on day 0 as previously described [3,13]. Parasitemia was assessed daily on Giemsa reagent-stained blood smears.

## Competing interests

The authors declare that they have no competing interests.

## Authors' contributions

All authors read and approved the final manuscript. RM, JS, JA-G performed all animal experiments, Biacore analysis, generation of Abs, and ELISAs. RM and JS contributed equally to the manuscript. SJD, EKF, AH and PHC provided parasite, human and/or rabbit serum samples and critiqued the manuscript. PKD constructed the pcDNA3.1/Hygro based human IgA1 expression vector. MvE & JvdW provided transgenic animals and important discussion. JMW provided laboratory facilities for vector production and wrote parts of the paper. RP conceived and designed the overall study, provided laboratory facilities, and wrote the paper.

## Supplementary Material

Additional file 1 **Characterization of CD89 transgenic mice**. FACS analysis of gated whole blood from CD89 negative or positive animals as assessed by PCR [19]. Gated neutrophils (R3, blue) and blood monocytes (R2, green) from positive animals used in this study bind PE-conjugated anti-human CD89 while those of CD89-negative animals do not. Anti-human IgA responses are provoked in mice passively administered with recombinant human IgA. Click here for file
