# Supplementary figures and images for: The generation and evaluation of recombinant human IgA specific for Plasmodium falciparum merozoite surface protein 1-19 (PfMSP119)
Source: BMC Biotechnol. 2011 Jul 22;11:77. doi: 10.1186/1472-6750-11-77 (PMC3199766; doi:10.1186/1472-6750-11-77)

Supp Fig 1

CD89 negative animals

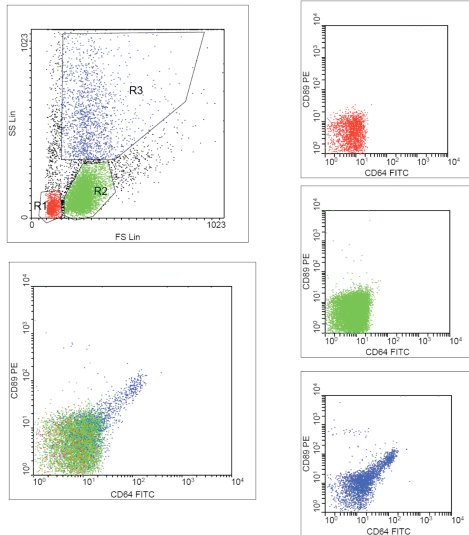

CD89 positive animals

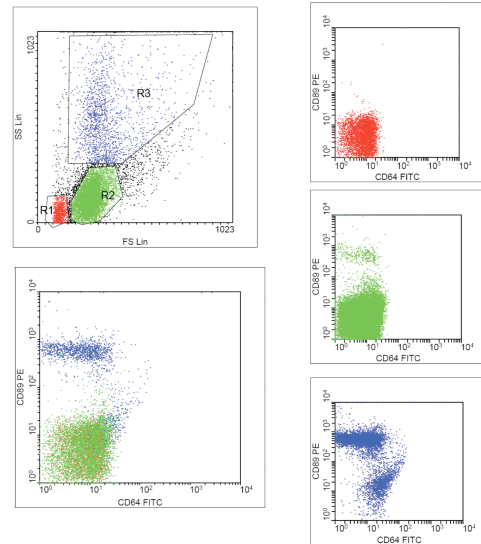

Supp Fig 2

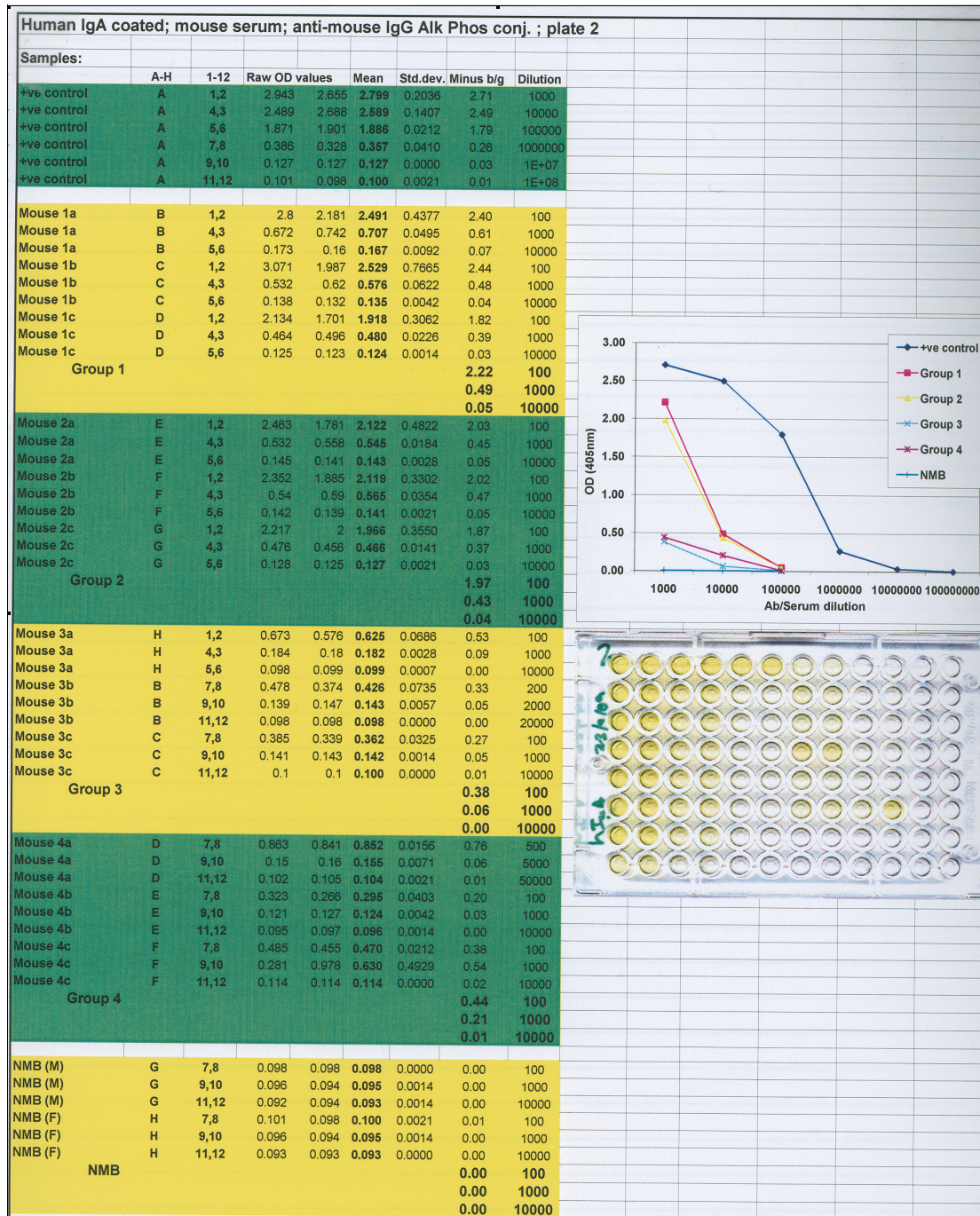

Supplement: Additional file 1 — Characterization of CD89 transgenic mice. FACS analysis of gated whole blood from CD89 negative or positive animals as assessed by PCR [19]. Gated neutrophils (R3, blue) and blood monocytes (R2, green) from positive animals used in this study bind PE-conjugated anti-human CD89 while those of CD89-negative animals do not. Anti-human IgA responses are provoked in mice passively administered with recombinant human IgA. [file 1472-6750-11-77-S1.PDF]
